# The Biological Product Agricultural Jiaosu Enhances Tomato Resistance to *Botrytis cinerea*

**DOI:** 10.3390/jof11120873

**Published:** 2025-12-10

**Authors:** Xue Lu, Nan Huang, Jing Ai, Lifang Fan, Lili Chen, Geng Meng, Jingna Liu

**Affiliations:** 1 School of Agriculture and Biotechnology, Shenzhen Campus of Sun Yat-sen University, No. 66, Gongchang Road, Guangming District, Shenzhen 518107, China; luxue5@alumni.sysu.edu.cn (X.L.); huangn58@mail2.sysu.edu.cn (N.H.); aijing3@mail2.sysu.edu.cn (J.A.); 2 Zijin Meteorological Bureau, Heyuan 517400, China; fanlifang01@126.com; 3 College of Science & Technology, Ningbo University, Ningbo 315300, China; chenlili2@nbu.edu.cn; 4 College of Horticulture, Henan Agricultural University, Zhengzhou 450002, China

**Keywords:** agricultural Jiaosu, *Botrytis cinerea*, antioxidant enzyme, antifungal activity, biological product, synergistic effects

## Abstract

Gray mold caused by *Botrytis cinerea* poses a major threat to tomato production worldwide. This study investigated the antifungal efficacy and defense-inducing potential of Agricultural Jiaosu (AJ), a fermented bioproduct derived from agricultural residues. In vitro, AJ exhibited strong inhibitory activity against *B. cinerea* (IC_50_ = 3.9%), primarily through acidic metabolites (pH < 4.2) that disrupted fungal membranes and suppressed antioxidant enzymes, while later-stage inhibition was maintained by *Acetobacter* populations (6.7 × 10^7^ copies μL^−1^) through competition for nutrients. In vivo, foliar application of 0.5% AJ significantly promoted tomato growth and enhanced resistance by stimulating antioxidant (SOD, CAT, POD) and defense-related (PAL, PPO) enzyme activities, reducing oxidative damage and lowering gray mold incidence by 55%. Collectively, AJ exerts a dual mode of action that combines direct pathogen suppression with activation of host systemic resistance. These results highlight AJ as a sustainable, residue-free biocontrol solution that offers an environmentally friendly alternative to chemical fungicides for effective management of gray mold in tomato cultivation.

## 1. Introduction

Tomato (*Solanum lycopersicum* L.) is a cornerstone of global agriculture, with annual production exceeding 180 million tons and a market value surpassing $100 billion [1]. Its cultivation is critical for food security, agricultural economies, and livelihoods in diverse regions globally. However, the sustainability of tomato production faces significant challenges from gray mold disease [2]. Gray mold caused by the necrotrophic fungal pathogen *Botrytis cinerea* can affect 10 to 20% of vegetable production, with severe cases reaching up to over 60% in some regions [3]. The incidence of gray mold in tomatoes ranges from 30% to 50%, resulting in yield reductions of 40% to 50% [4]. In severe cases, it can lead to complete crop loss, making it a major limiting factor in tomato production and resulting in worldwide annual losses estimated at $10 billion to $100 billion [5]. Ranked among the ten most destructive plant pathogens worldwide, *B. cinerea* infects over 1400 plant species [6]. This pathogen exhibits remarkable adaptability due to its highly plastic nature [7]. Its asexual conidia possess thick cell walls endowing resistance to dehydration and ultraviolet light, while its melanized hyphae can survive dormant under extreme temperatures [8]. These characteristics enable *B. cinerea* to thrive in diverse environmental conditions, significantly complicating efforts to control its spread and rendering conventional control strategies increasingly inadequate [9].

Chemical fungicides, which account for 10% of global fungicide applications, are the primary method for controlling gray mold [5,10]. Despite their efficacy, prolonged reliance on chemical fungicides poses substantial risks, such as environmental pollution, residual health threats, evolution of pathogen resistance, and loss of biodiversity [11]. Therefore, relying exclusively on chemical pesticides is not sustainable for plant disease management. There is a pressing need for alternative control solutions that are both effective against gray mold and safe for human health and the environment.

Given the drawbacks of chemical pesticides, biological control has emerged as a promising alternative owing to its safety, absence of residues, sustainability, and lower risk of resistance development [12]. Botanical fungicides and biological agents have become the main alternatives to synthetic fungicides. Certain phytochemical fungicides, such as terpenoids, chitosan, and essential oils, not only inhibit *B. cinerea* growth but also exhibit broad-spectrum fungicidal activity [13]. Similarly, microbial biological agents, notably *Bacillus* spp. [14], *Pseudomonas* spp. [15], *Lactobacillus* spp. [16], and *Penicillium* spp. [17], represent another major focus as potent antagonistic microorganisms. Nevertheless, both alternatives face significant challenges including complex production processes, high costs, and inconsistent field efficacy, which collectively limit their widespread adoption. To overcome these constraints and realize the full potential of biological control, future efforts must prioritize developing efficient, low-cost, and environmentally sustainable products rich in both antifungal compounds and robust antagonistic microbes.

Agricultural Jiaosu (AJ), a biological product derived from fermented agricultural byproducts, is a complex ecosystem rich in organic acids (e.g., acetic, lactic, propionic) and beneficial microorganisms (e.g., *Acetobacter*, *Lactobacillus*), characterized by a pH below 4 [18]. These organic acids inhibit fungal growth primarily by acidifying the environment; additionally, their undissociated forms penetrate the fungal cell membrane owing to their inherent lipophilicity [19]. Inside the cell, these acids dissociate, disrupting the proton gradient, acidifying the cytoplasm, and inhibiting metabolic processes, including ATP production and enzyme activities (e.g., decarboxylase, catalase) [20,21]. Additionally, AJ’s microbial components suppress pathogens through competition for nutrients, direct antimicrobial activity, and induction of plant systemic resistance [22]. Previous studies have demonstrated AJ’s efficacy in controlling *Fusarium* spp. (61.43% reduction in root rot incidence in *Astragalus* [23]) and *B. cinerea* (IC_50_ value of 9.24% [18]) in non-solanaceous crops. However, its application in tomato production and specific mechanisms underlying its activity against *B. cinerea* remain underexplored. The lack of systematic evaluation of the growth-promoting effects of AJ on tomato plants and its antifungal mechanisms hinders the optimization of its application in tomato production systems.

This study aims to: (1) characterize AJ’s in vitro antifungal activity against *B. cinerea*; (2) elucidate AJ’s inhibitory mechanisms through ultrastructural and biochemical analyses; (3) evaluate AJ’s effects on tomato growth and gray mold suppression. By addressing these gaps, the study provides a theoretical and practical foundation for AJ’s application as a sustainable biocontrol strategy in tomato production.

## 2. Materials and Methods

### 2.1. Preparation of Agricultural Jiaosu (AJ) and Pathogen Cultivation

The AJ was prepared at the Biomass Engineering Center, College of Agronomy and Biotechnology, China Agricultural University (Beijing, China). The raw material consisted of fresh stem and leaf waste from four medicinal plants (*Astragalus*, *Scutellaria baicalensis*, *Radix et Rhizoma Ginseng*, and *Radix Bupleurum*) mixed in equal fresh weight proportions (1:1:1:1). The plant mixture was combined with brown sugar and water (3:1:10 ratio) and fermented anaerobically in 5 L sealed glass containers for 90 days at 30 ± 2 °C in a temperature-controlled chamber to produce the final AJ product.

The *B. cinerea* strain B05.10 was obtained from Henan Agricultural University. This strain is the reference strain for the *Botrytis* research community, with a fully sequenced genome [24]. It was cultured on potato dextrose agar (PDA) medium at 25 °C in darkness for 5 days. To promote sporulation, the fungus was subsequently incubated on PDA under identical conditions for an additional 10 days. Conidia were harvested by rinsing the culture plates with sterile distilled water, filtering the suspension through three layers of lens paper, and centrifuging at 5000× *g* for 5 min. The supernatant was discarded, and the pellet was resuspended in 1 mL of sterile distilled water. Conidium concentration was determined using a hemocytometer with a 7 μL sample aliquot under a light microscope and adjusted to 1 × 10^6^ CFU/mL for experimental use.

### 2.2. Characterization of AJ

The pH of AJ was determined using a digital pH meter (model a-AB33PH ZH, OHAUS, Shanghai, China). Organic acid composition (formic, acetic, propionic, butyric, and lactic acids) was analyzed by high-performance liquid chromatography (HPLC; LC-20A, Shimadzu, Kyoto, Japan). Samples were centrifuged and the supernatant was filtered through 0.22 μm aqueous phase filters prior to injection. Quantification was achieved using external standard calibration curves. A series of standard solutions at known concentrations (ranging from 0.05 to 1.0 g/L) for each acid were analyzed to establish linear calibration curves (R^2^ > 0.99), ensuring quantitative accuracy. The analysis was performed using an HPLC system equipped with a C18 reverse-phase column. Analytical conditions included a UV detection wavelength of 204 nm, a mobile phase of 0.272 mM H_2_SO_4_ (pH ≈ 2.1) at a flow rate of 0.6 mL/min, and isothermal column operation at 40 °C for 30 min. Characterization of volatile organic compounds (VOCs) was performed using gas chromatography–mass spectrometry (GC–MS; Agilent 7890A GC/5975C MS) by Hangzhou Yanqu Information Technology Co., Ltd. (Hangzhou, China). The analysis followed the company’s standard operational protocol, which included solid-phase microextraction (SPME) for sample preparation and qualification against the NIST11 mass spectral library. This validated method ensures the reliability and reproducibility of the qualitative profiling of VOCs [25]. Microbial community analysis was performed through high-throughput sequencing on an Illumina noveseq 6000 platform by Personalbio Company (Shanghai, China). The primers listed in Table 1 were used, and the resulting sequences were classified taxonomically against the SILVA (Release 138) [26] and UNITE (Release 9.0) [27] databases for bacteria and fungi communities, respectively. A detailed summary of the identified microbial taxa, including taxonomic classification, confidence, and representative sequencing reads, is provided in Supplementary Table S1.

### 2.3. In Vitro Antifungal Activity Assays

The inhibitory effect of AJ on *B. cinerea* was assessed through agar diffusion assays [17]. AJ was incorporated into PDA medium at seven concentrations (1%, 2.5%, 5%, 7.5%, 10%, 15%, and 25%), PDA without AJ as the control. For each concentration, 20 mL of medium was poured into 9 cm diameter Petri dishes, with three replicate plates (n = 3) per treatment. A 5-mm mycelial plug from 5-day-old *B. cinerea* cultures was then aseptically transferred to the center of each plate. Sealed plates were incubated at 25 °C in darkness for 120 h, with colony diameters measured every 24 h using the crisscross method [28]. For quantitative analysis, a linear regression was performed with AJ concentration as the independent variable (x) and colony diameter at 120 h as the dependent variable (y). The half-maximal inhibitory concentration (IC_50_) was determined by substituting half the control colony diameter into the regression equation [29]. The inhibition percentage was calculated as:

(1) $$Inhibition\left(\%\right)=\left(Fck-FAJ\right)/\left(Fck-F0\right)\times 100$$

For spore germination and viability assessment, the treatment contained 1 mL of *B. cinerea* conidium suspension (1 × 10^5^ CFU mL^−1^) with 100 μL of 0.1% glucose solution and 1 mL of AJ at varying concentrations (1%, 2.5%, 5%, 7.5%, 10%, 15%). The control contained 100 μL of 0.1% glucose solution and 1 mL sterile water. All treatments (n = 3 biological replicates per concentration) were incubated at 25 °C in darkness for 12 h. Spore germination was assessed by examining 50 μL aliquots on slotted slides under light microscopy. Samples were incubated with FDA/PI fluorescent dyes (490/520 nm and 535/615 nm) at 37 °C for 15 min for dual staining. After PBS (pH 7.4) washing, spore viability was determined using an inverted fluorescence microscope.

### 2.4. Mechanism Analysis of AJ Action

The antifungal contribution of AJ’s microbial components against *Botrytis cinerea* was evaluated using four treatments (n = 3 biological replicates): 5% raw AJ, 5% sterilized AJ, 5% microbial fraction of AJ, and control (CK). Following the method described in Section 2.3, inhibition rates were determined after 5 days of incubation. The relative contribution of individual components to pathogen suppression was determined through the following equation [23]:

(2) MCR%=IR/IR×100

(3) OCR%=1−MCR%

In this formula: OCR represents the contribution percentage of non-microbial components, IRs denotes the inhibition percentage of centrifuged AJ treatment, IR indicates the total inhibition percentage of AJ treatment, MCR stands for the microbial contribution percentage in AJ. Dynamic profiling of AJ active components: the experiment utilized 100 mL of potato-dextrose broth (PDB) medium, with 2.5% AJ dilution as the treatment and pure PDB as the control (CK). Each flask was inoculated with three 5-mm diameter *B. cinerea* mycelial plugs (n = 3 biological replicates) and incubated at 25 °C with 175 rpm shaking in darkness. Samples were collected at 0, 24, 48, 60, and 72 h for pH and organic acid analysis. Subsets (0, 24, 60, and 72 h) were processed for DNA extraction, targeting *Lactobacillus*, Acetobacter, and *B. cinerea* quantification via quantitative PCR (qPCR). DNA was extracted using a commercial kit (M5635-02, Omega Bio.tek, Norcross, GA, USA). The qPCR setup (20 μL final volume) was prepared with: 10 μL 2× SYBR Green PCR mix and 0.4 μL each of forward and reverse primers (Table 2). Thermal cycling conditions comprised: initial denaturation (95 °C, 5 min); 40 cycles of denaturation (95 °C, 15 s) and annealing/extension (60 °C, 30 s).

Scanning electron microscope (SEM) analysis: mycelial segments of *Botrytis cinerea* (5 × 5 mm^2^) underwent fixation in 2.5% glutaraldehyde at 4 °C for 12 h, then were rinsed three times with 0.1 M phosphate buffer (PB, pH 7.4). Post-fixation was performed with 1% osmium tetroxide (OsO4) in darkness for 2 h, with subsequent buffer washes. After dehydration through an ethanol gradient series, samples were paraffin-embedded. Mesophyll cell ultrastructure was examined using a Hitachi SU-7800 field emission scanning electron microscope (Tokyo, Japan).

Potato dextrose broth (PDB, 100 mL) was used to prepare AJ solutions at 2.5% and 7.5% concentrations, with pure PDB serving as the control (CK). Each treatment flask was inoculated with 1 g of *B. cinerea* mycelium (n = 3 biological replicates) and incubated at 25 °C with 175 rpm shaking in darkness. Samples were collected at 0, 1, 2, 3, and 4 h for measurement of electrolyte conductivity and optical density at 260 nm (OD260). Moreover, samples were collected at 2 h for analysis. The malondialdehyde (MDA) content and enzyme activities (SOD, CAT, POD; n = 3 biological replicates).

MDA content was determined according to established methods [32]. Briefly, 0.2 g of leaf tissue was homogenized in 0.1% trifluoroacetic acid (TFA), and 1 mL of the homogenate was reacted with 4 mL of a solution containing 20% TFA and 0.5% 2-thiobarbituric acid (TBA). Absorbance was measured at 532 nm and 600 nm using a microplate reader, with the reading at 600 nm serving as a reference for baseline correction.

Enzyme activity measurements were performed with commercially available test kits (Norminkoda Biotechnology, Nanjing, China) following the manufacturer’s instructions:

- Superoxide dismutase (SOD; Cat# A001-1-2)
- Catalase (CAT; Cat# A007-1-1)
- Peroxidase (POD; Cat# A084-3-1)

### 2.5. Plant Treatment Experiments

Tomato seeds (*Solanum lycopersicum* L. cv. M82) were germinated in Petri dishes lined with double-layer filter paper and incubated at 28 ± 1 °C in complete darkness for 72 h. Germinated seeds were transplanted into a growth medium consisting of peat: vermiculite: perlite (3:1:1, *v*/*v*). After the development of secondary true leaves, seedlings were transplanted into plastic containers (21.5 × 16 × 10 cm) containing half-strength Hoagland’s nutrient solution for experimental treatments. Plants were grown under controlled environmental conditions: 25/20 °C day/night temperature, 70–80% relative humidity, and a 16/8 h light/dark photoperiod with a photosynthetic photon flux density of 300 μmol·m^−2^·s^−1^.

At the third true leaf stage, the experimental treatments commenced. Seedlings were randomly divided into two groups arranged in a completely randomized design: the control group (CK) and the Agricultural Jiaosu treatment group (AJ). Each treatment group consisted of 30 plants. To maintain normal plant growth, all plants were irrigated with half-strength Hoagland’s nutrient solution every five days. For foliar spray treatments, the AJ group received a 0.5% (*v*/*v*) AJ solution, while the CK group received an equal volume of sterile distilled water. Both groups were sprayed once a week at 8:00 AM, with a volume of 5 mL per plant. This treatment regimen was sustained for a total of 28 days.

Following the 28-day treatment period, parallel analyses for disease resistance and growth physiology were conducted. For disease resistance assessment, twenty plants (n = 20) from each group were subjected to *B. cinerea* infection experiments. These plants were uniformly sprayed with a spore suspension (10^6^ CFU mL^−1^). As a protective treatment, the AJ group received a foliar application of 0.5% AJ solution 12 h post-inoculation. Pathogenicity was evaluated after 10 days of incubation using a standardized disease index scale: 0: No symptoms; 1: Slight wilting with normal growth; 2: Necrotic leaf spots, root lesions, and stunted growth; 3: ≥2/3 plant wilted/necrotic with diseased roots; 4: Complete plant necrosis [33]. The disease index was calculated using the formula:

(4) $$Disease\;index=\frac{\Sigma (Disease\;scale\times Number\;of\;plants\;at\;that\;scale)}{Total\;number\;of\;plants\times Highest\;disease\;scale}\times 100$$

This provides a standardized measure ranging from 0 (all plants completely healthy) to 100 (all plants at the most severe disease level). Additionally, a detached leaf assay was conducted to quantify infection severity. Leaves were harvested from three different plants per treatment to serve as three biological replicates (n = 3). For inoculation, a 5-mm diameter mycelial plug, taken from the actively growing margin of a 5-day-old *B. cinerea* B05.10 culture on PDA, was placed onto each leaf. To facilitate infection, the leaves were either lightly wounded at the inoculation site with a sterile needle or the plug was placed on the uninjured leaf surface. The inoculated leaves were then incubated in a humid chamber at 25 °C. After 60 h, the lesion area around each plug was measured, and the infection percentage was calculated relative to the total leaf area.

Concurrently, growth and physiological analyses were performed on the remaining plants not used in the in vivo infection assays. From these, growth parameters (plant height and stem diameter) were measured on six individual plants (n = 6). For the analysis of physiological indices, the third and fourth true leaves were harvested, immediately frozen in liquid nitrogen, and ground to a fine powder. Leaf tissues from the remaining plants were pooled to form one biological replicate, with three independent replicates (n = 3) analyzed per treatment. (MDA) content and the activities of antioxidant enzymes (SOD, CAT, POD) were analyzed following the methods detailed in the “Mechanism Analysis of AJ Action” section. Additionally, the activities of phenylalanine ammonia-lyase (PAL) and polyphenol oxidase (PPO) were determined using commercial test kits from Norminkoda Biotechnology (Nanjing, China), specifically PAL (PAL; Cat# A137-1-1) and PPO (PPO; Cat# NMKD0101), according to the manufacturer’s protocols. including malondialdehyde (MDA) content and antioxidant enzyme activities, following the method described in Section 2.4; leaf tissues (the third and fourth true leaves) were harvested and pooled from the remaining plants to constitute one biological replicate, with three independent replicates (n = 3) analyzed per treatment.

### 2.6. Data Analysis

Data were statistically analyzed using GraphPad Prism software (version 9.0.0) and SPSS (version 2023). For comparisons between two groups, an unpaired Student’s t-test was employed. For comparisons among multiple groups, a one-way analysis of variance (ANOVA) was performed, followed by Tukey’s post hoc test. The distribution of disease severity over time was assessed using the Chi-square test of independence. The significance level was set at *p* < 0.05 for all tests.

## 3. Results

### 3.1. Compositional Characteristics of AJ

The AJ had a pH value of 3.21. Acetic acid was the dominant organic acid, with concentrations 3.58, 1.74, and 4.56 times higher than those of propionic, butyric, and lactic acids, respectively (Table 3).

Additionally, AJ’s volatile organic compounds (VOCs) included alcohols, esters, phenols, ketones, aldehydes, and ethers, with L-α-terpineol, phenylethyl alcohol, and D-limonene constituting 6.78%, 0.79%, and 0.49% of the composition, respectively (Table 4).

**Figure 1:**
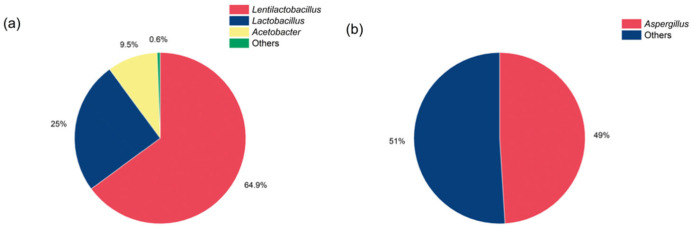
Microbial community structure of AJ at the genus level. (**a**) Bacterial community. (**b**) Fungal community.

High-throughput analyses were employed to evaluate both bacterial and fungal diversity in AJ, revealing significantly greater bacterial diversity compared to fungal diversity. The dominant bacterial genera were *Lentilactobacillus*, *Lactobacillus*, and *Acetobacter* (Figure 1a), while the predominant fungal genus was *Aspergillus* (Figure 1b).

### 3.2. Antifungal Activity of AJ Against Botrytis cinerea In Vitro

**Figure 2:**
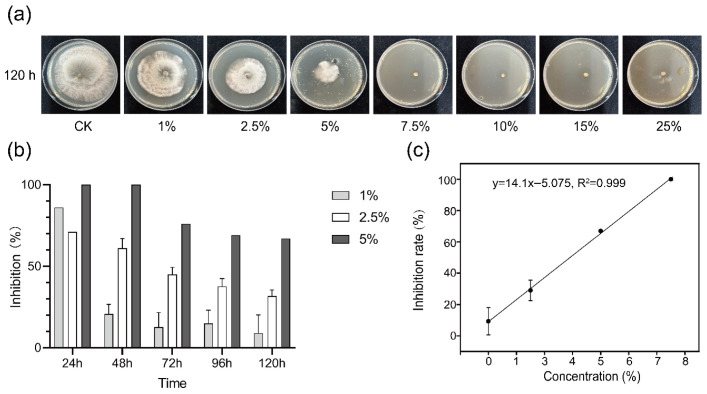
Antifungal activity. (**a**) Antifungal activity of different doses of AJ at 120 h. (**b**) Inhibition rates of 1%, 2.5%, 5% dosage of AJ at different periods. (**c**) IC_50_ of AJ against *Botrytis cinerea*.

We utilized the plate dilution assay on agar medium to examine the antifungal potential of AJ against *B. cinerea*. After 120 h, CK exhibited the largest colony diameter (78 mm; *p* < 0.05), indicating significant inhibition of *B. cinerea* hyphal growth by AJ (Figure 2a). The inhibition rate was positively correlated with AJ concentration, with higher concentrations demonstrating stronger inhibitory effects. At AJ concentrations above 7.5%, pathogen growth was completely inhibited (100% inhibition rate) at 120 h (Figure 2b). At 5% AJ, the inhibition rate exceeded 60% at 120 h, with complete suppression of mycelial growth within 48 h, indicating effective inhibition (Figure 2b). Furthermore, 2.5% AJ was particularly effective in inhibiting mycelial growth during the initial 48 h. According to the fitting equation in Figure 2c, the IC_50_ value of AJ against *Botrytis cinerea* was determined to be 3.9%.

**Figure 3:**
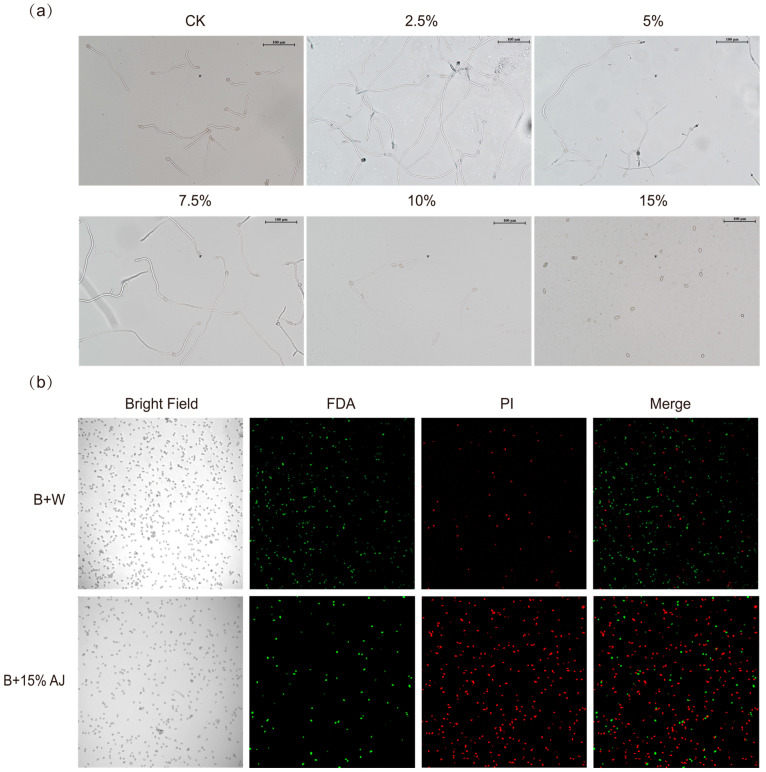
Effect of AJ on *Botrytis cinerea*’s conidium germination and viability. (**a**) Effects of various AJ concentrations on *B. cinerea*’s conidium germination at 12 h (scale bar = 100 μm). (**b**) Effects of 15% AJ on *B. cinerea*’s conidium viability at 12 h. Bright Field: phase contrast images; FDA: fluorescein diacetate signal; PI: propidium iodide signal; Merge: merged FDA and PI signals; B + W: *B. cinerea*’s spore suspension + sterilized water; B + 15% AJ: *B. cinerea’s* conidium suspension + 15% AJ.

Various concentrations of AJ were applied to *B. cinerea* conidium cultures. After 12 h, conidium germination was observed under a microscope. As shown in Figure 3a, germination was facilitated at AJ concentrations below 10%, while 15% AJ completely inhibited germination compared to CK. Additionally, a double staining method using fluorescein diacetate (FDA) and propidium iodide (PI) was employed to assess the viability of *B. cinerea’s* conidium. According to Figure 3b, 15% AJ also effectively inhibited the viability of the conidia.

#### Contribution of AJ Components Against *Botrytis cinerea*

**Figure 4:**
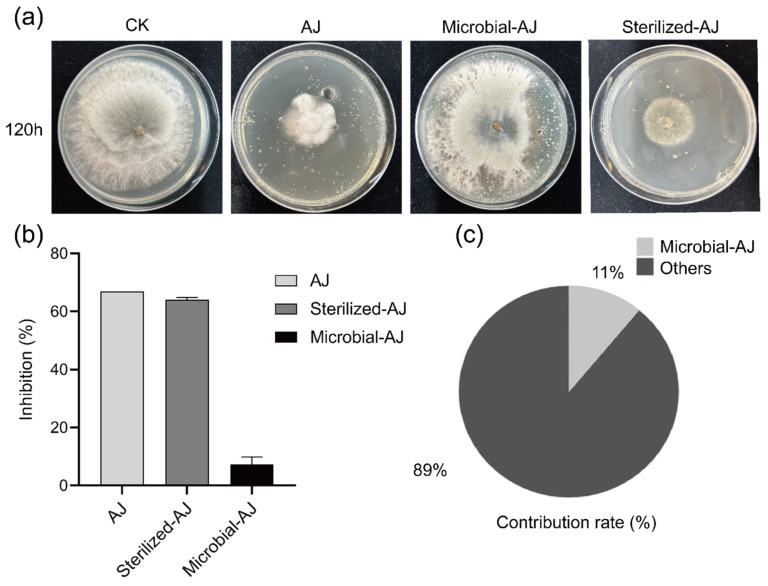
Antifungal activity of AJ components against *Botrytis cinerea* at 120 h. (**a**) Antifungal activity of Sterilized-AJ and Microbial-AJ compared to CK. (**b**) Inhibition rates of Sterilized-AJ and Microbial-AJ. (**c**) Relative contribution of Sterilized-AJ and Microbial-AJ to inhibition. CK: control (sterile water); AJ: 5% Agricultural Jiaosu; Microbial-AJ: microbial fraction of 5% AJ; Sterilized-AJ: sterilized fraction of 5% AJ.

To identify the primary antifungal components of AJ, 5% AJ was separated into sterilized (Sterilized-AJ) and microbial (Microbial-AJ) fractions, and their antifungal effects against *B. cinerea* were assessed at 120 h. Figure 4a shows that Sterilized-AJ reduced colony diameter more effectively than Microbial-AJ compared to CK. Additionally, Figure 4b,c indicated that Sterilized-AJ exhibited 9.14 times greater inhibition and 8.09 times greater contribution to inhibition than Microbial-AJ (*p* < 0.05). These results suggest that the sterilized fraction, primarily organic acids, is the main contributor to AJ’s antifungal activity against *B. cinerea*.

### 3.3. Mechanism of Botrytis cinerea Inhibition by AJ In Vitro

#### 3.3.1. Temporal Dynamics of the Interaction Between AJ and *Botrytis cinerea*

**Figure 5:**
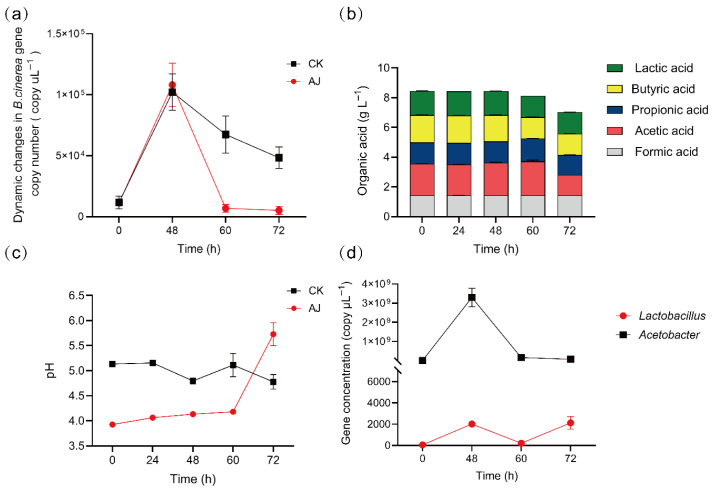
Dynamic changes in AJ components during *Botrytis cinerea* inhibition over 0–72 h. (**a**) Changes in the gene concentration of *B. cinerea* at 0~72 h. (**b**) Dynamic changes in *Lactobacillus* and *Acetobacter* gene copy number during AJ treatment. (**c**) pH changes in AJ-treated samples compared to CK. (**d**) Changes in formic, acetic, propionic, butyric, and lactic acid concentrations during AJ treatment.

To investigate the antifungal mechanisms of Agricultural Jiaosu (AJ), qPCR quantified *Acetobacter*, *Lactobacillus*, and *Botrytis cinerea* in AJ-treated samples over time. AJ treatment significantly reduced *B. cinerea* gene copy numbers (*p* < 0.05), with CK exhibiting 8.18-fold higher pathogen levels than AJ-treated samples. The *B. cinerea* gene copy number in AJ-treated samples rapidly decreased from 1.08 × 10^5^ copy μL^−1^ at 48 h to 6.97 × 10^3^ copy μL^−1^ at 60 h (Figure 5a). Simultaneously, the dynamic changes in organic acids and pH value in the AJ-treated samples were quantified, revealing a rapid pH decrease compared to CK due to organic acid accumulation (Figure 5c). As organic acids were depleted, the pH increased from 4.18 at 60 h to 5.73 at 72 h, a 37% rise (Figure 5b,c). Furthermore, the gene abundance of *Acetobacter* had a peak of 3.29 × 10^9^ copy μL^−1^ at 48 h, followed by a downward trend in the later stages, though the lowest value remained at 6.69 × 10^7^ copy μL^−1^, which was 1.26 × 10^4^ times that of *B. cinerea* (Figure 5d).

#### 3.3.2. Effect of AJ on the Ultrastructure of *Botrytis cinerea* Mycelium

**Figure 6:**
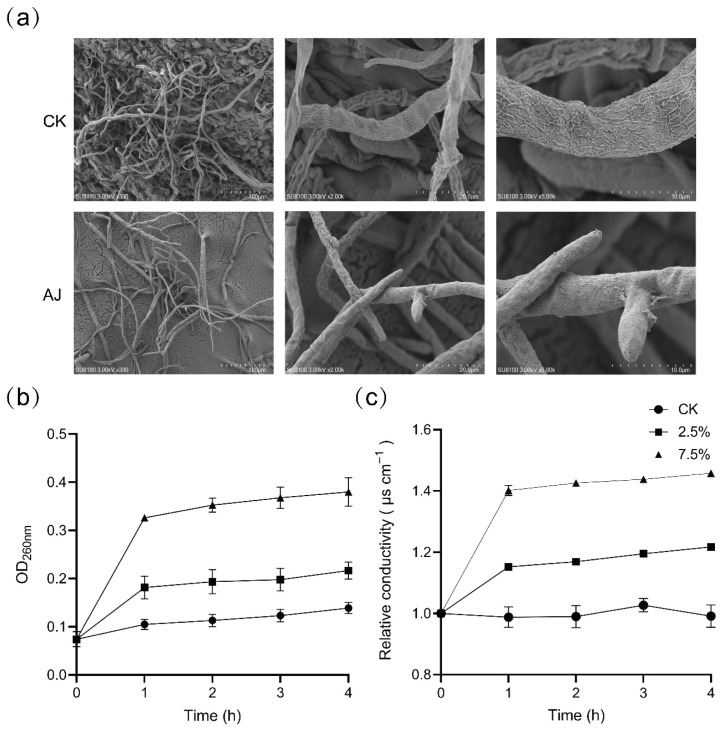
Effects of AJ on *Botrytis cinerea* mycelium ultrastructure and biochemistry over 0–4 h. (**a**) Ultrastructure of *B. cinerea* mycelium under SEM. T (**b**,**c**) Dynamic changes of OD_260 nm_ and relative conductivity of *B. cinerea* at 0~4 h. CK: control (sterile water); AJ: Agricultural Jiaosu.

The ultrastructure of *B. cinerea* mycelium was examined using TEM. Figure 6a illustrates that AJ reduced *B. cinerea* mycelial density and disrupted cell wall integrity. Concurrently, the OD_260 nm_ values and relative conductivity of the mycelium were measured. Compared to CK, OD_260 nm_ values for mycelium treated with 2.5% and 7.5% AJ increased by 1.90- and 4.10-fold, respectively (*p* < 0.05; Figure 6b), while relative conductivity increased by 16% and 47% after 4 h (*p* < 0.05; Figure 6c).

#### 3.3.3. Effect of AJ on Antioxidant Enzyme Activity and MDA Content in *Botrytis cinerea*

**Figure 7:**
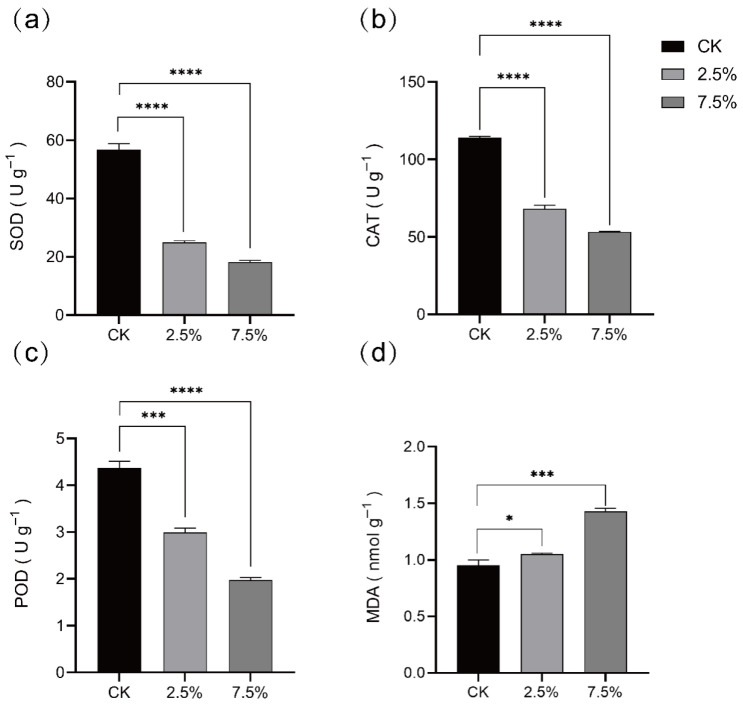
Antioxidant enzyme activity and MDA content of *Botrytis cinerea*. (**a**) SOD activity. (**b**) CAT activity. (**c**) POD activity. (**d**) MDA content. The antioxidant enzyme activity and MDA of *B. cinerea* were determined after 2.5% and 7.5% AJ treatment for 2 h; a Student’s t-test was used for significant analysis; *, *p* < 0.05; ***, *p* < 0.001; ****, *p* < 0.0001; error bars indicate S.D. (n = 3). CK: control group without AJ; 2.5%: 2.5% AJ; 7.5%: 7.5% AJ; SOD: superoxide; CAT: catalase; POD: peroxidase; MDA: malondialdehyde.

To assess AJ’s impact on fungal stress tolerance, some stress-resistance indices, including SOD, POD, and CAT, were analyzed as biomarkers. The results showed that *B. cinerea* treated with AJ exhibited significantly lower activities of SOD, CAT, and POD compared to those treated with PDB (Figure 7a–c) (*p* < 0.001). Additionally, AJ treatment led to an increase in MDA in *B. cinerea* (Figure 7d).

### 3.4. Effect of Agricultural Jiaosu (AJ) on the Incidence of Botrytis cinerea in Tomato Plants

**Figure 8:**
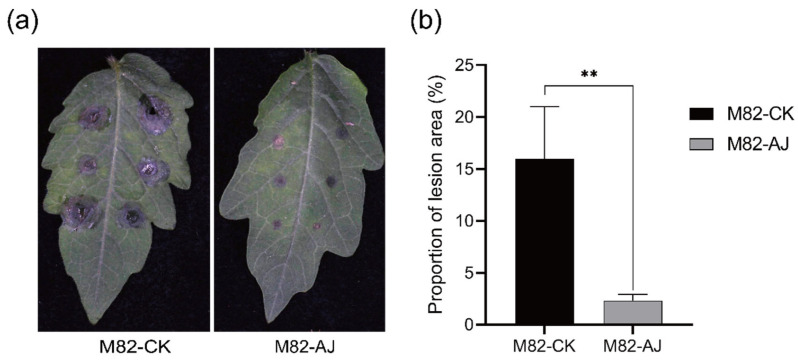
The incidence of gray mold. (**a**) The picture of the lesions. (**b**) The proportion of the lesion area. Leaves were inoculated with a *B. cinerea* mycelial plug and incubated at 25 °C for 60 h. A Student’s *t*-test was used for significant analysis; **, *p* < 0.01; error bars indicate S.D. (n = 3).

Visual assessment of disease symptoms revealed a marked reduction in lesion development on AJ-treated leaves compared to the control (Figure 8a), corroborated by a significant decrease in lesion area proportion (*p* < 0.01; Figure 8b). Quantitative analysis affirmed this protective effect, showing that AJ treatment restricted 55% of plants to mild disease severity (Scale 0–2) and reduced the disease index by 55.3% (from 95.0 to 42.5) (Table 5). A Chi-square test confirmed a highly significant alteration in disease severity distribution following AJ treatment (χ^2^ = 24.56, *p* < 0.001).

**Figure 9:**
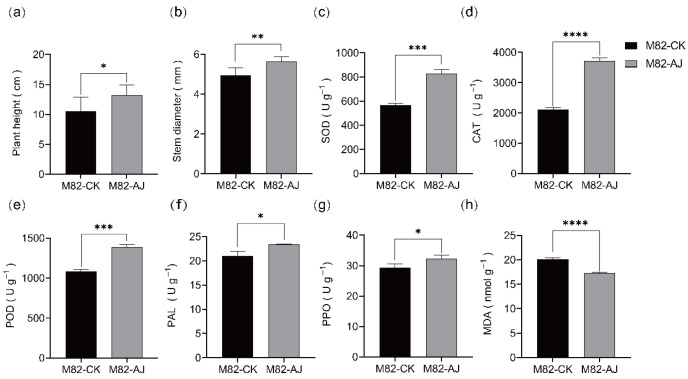
Effect of AJ on physiological and biochemical characteristics of tomato plants. (**a**) Plant height. (**b**) Stem diameter. (**c**) SOD activity. (**d**) CAT activity. (**e**) POD activity. (**f**) PAL activity. (**g**) PPO activity. (**h**) MDA content. Tomato plants were sprayed with 0.5% AJ (5 mL per plant) or sterile water (control, CK) every 7 days at 8:00 AM, and physiological and biochemical indicators were measured after 28 days. A Student’s t-test was employed for significant analysis; ns, no significant difference; *, *p* < 0.05; **, *p* < 0.01; ***, *p* < 0.001; ****, *p* < 0.0001; error bars denote ± S.D. (n = 3). CK: control group (sterile water); AJ: 0.5% Agricultural Jiaosu; SOD: superoxide dismutase; CAT: catalase; POD: peroxidase; PAL: phenylalanine ammonia-lyase; PPO: polyphenol oxidase; MDA: malondialdehyde.

To investigate how AJ enhances tomato plant resistance, this study evaluated the physiological and biochemical characteristics of AJ-treated plants. The results showed that AJ significantly increased plant height from 10.50 cm to 13.17 cm (a 25.4% increase; *p* < 0.05; Figure 9a) and stem diameter from 4.94 mm to 5.65 mm (a 14% increase; *p* < 0.05; Figure 9b). Additionally, the activities of key enzymes in AJ-treated plants were significantly elevated compared to the control (CK): superoxide dismutase (SOD) increased from 569.5 U/g FW to 829.6 U/g FW (27.5% increase; *p* < 0.05; Figure 9c), catalase (CAT) from 2114 U/g FW to 3716 U/g FW (75.8% increase; *p* < 0.05; Figure 9d), and peroxidase (POD) from 1086 U/g FW to 1385 U/g FW (45.7% increase; *p* < 0.05; Figure 9e). The defense-related enzymes phenylalanine ammonia-lyase (PAL) and polyphenol oxidase (PPO) also showed increased activities, from 21.02 U/g FW to 23.37 U/g FW (10.2% increase; *p* < 0.05; Figure 9f) and from 29.3 U/g FW to 32.23 U/g FW (10% increase; *p* < 0.05; Figure 9g), respectively. Furthermore, malondialdehyde (MDA) content in AJ-treated plants significantly decreased from 20.14 nmol/g FW to 17.3 nmol/g FW (a 16.4% reduction; *p* < 0.0001; Figure 9h), indicating mitigated oxidative damage.

## 4. Discussion

This study demonstrates that Agricultural Jiaosu (AJ) effectively controls tomato gray mold through a synergistic dual mechanism: directly suppressing *B. cinerea* and enhancing plant systemic resistance. The integration of in vitro and in vivo experiments provides comprehensive insights into AJ’s efficacy and underlying mechanisms, supporting its potential as an eco-friendly biocontrol strategy.

### 4.1. Direct Inhibition: Targeted Suppression of Botrytis cinerea

AJ directly suppressed *B. cinerea* through its acidic substances and microbial components, as demonstrated by in vitro assays. At 7.5% concentration, AJ completely inhibited mycelial growth after 120 h, while 15% AJ prevented spore germination and viability (*p* < 0.05; Figure 2a,b and Figure 3a,b). The IC_50_ value of 3.9% (Figure 2c) highlights AJ’s potent antifungal activity, surpassing previous reports (e.g., IC_50_ of 9.24% by Zhang et al., 2020 [18]). The antifungal efficacy was primarily driven by AJ’s acidic substances, contributing 89% to the inhibition compared to 11% from microbial components (*p* < 0.05; Figure 4c). This dual-action acidic disruption and microbial antagonism produces a synergistic effect, as described below.

AJ’s low pH (3.21; Table 3) and high organic acid content (e.g., acetic acid at 27.31 g L^−1^) create a collectively unfavorable environment for *B. cinerea*. During the initial 0–60 h, the pH remained below 4.18 (Figure 5c), with stable organic acid levels (Figure 5d), leading to intracellular acidification. Undissociated organic acids (e.g., acetic, propionic) penetrate fungal membranes, dissociate intracellularly, and disrupt proton gradients, overwhelming H^+^-ATPase pumps [34,35,36,37]. This process increased relative conductivity by 47% and OD_260 nm_ leakage by 4.1-fold at 7.5% AJ (*p* < 0.01; Figure 6b,c), confirming membrane permeabilization and cellular content leakage. Transmission electron microscopy (TEM) revealed compromised mycelial integrity (Figure 6a), consistent with acid-induced lipid phase transitions [38]. Additionally, organic acids suppressed fungal antioxidant defenses, reducing SOD, CAT, and POD activities by 28–54% (*p* < 0.001; Figure 7a–c) and increasing MDA content by 50% (*p* < 0.001; Figure 7d), indicating oxidative stress and redox system collapse [39]. Within this acid-dominated inhibitory environment, volatile organic compounds (VOCs) such as L-α-terpineol (6.78%; Table 4) further enhanced antifungal efficacy through gas-phase diffusion, causing severe damage to hyphal ultrastructure [40,41]. From 60–72 h, as organic acids were depleted and pH rose to 5.73 (Figure 5c), microbial antagonism became more prominent. High-throughput sequencing and qPCR revealed *Acetobacter* dominance (6.69 × 10^7^ copies μL^−1^ at 60 h; Figure 5d), outcompeting *B. cinerea* (6.97 × 10^3^ copies μL^−1^; Figure 5a) by four orders of magnitude. This nutrient competition, coupled with antagonistic metabolites from *Acetobacter* and *Lactobacillus* [13], sustained inhibition after acid depletion. Unlike Gao et al. (2022) [23], who emphasized microbial dominance, our findings highlight acidic substances as the primary inhibitory factor, with microbes playing a secondary but synergistic role. This initial, temporally dynamic, acid-driven suppression followed by microbial competition explains AJ’s sustained efficacy against *B. cinerea*.

### 4.2. Indirect Inhibition: AJ-Induced Systemic Resistance in Tomato

In addition to its direct inhibitory effects, AJ significantly enhanced tomato resistance to *B. cinerea* by promoting plant growth and activating defense pathways. After 28 days of foliar AJ application, tomato plants exhibited a 25.4% increase in height and a 14% increase in stem diameter compared to the control (*p* < 0.05; Figure 8a,b). These growth-promoting effects likely result from AJ’s organic acids (e.g., acetic and propionic acids; Table 2), which increase the solubility of nutrients such as K, Fe, Zn, and P [42]. Improved nutrient availability fosters robust root systems and microbial symbiosis [43], which in turn enhances overall plant vigor. This vigor, characterized by thicker stems and denser root systems, physically impedes *B. cinerea* hyphal invasion and forms a primary defense barrier [44].

AJ treatment also markedly upregulated key antioxidant and defense enzymes. SOD, CAT, and POD activities increased by 27.5%, 75.8%, and 45.7%, respectively (*p* < 0.01; Figure 9c–e), effectively scavenging reactive oxygen species (ROS) generated during pathogen infection. Excess ROS disrupts redox balance and causes membrane peroxidation [45]. Accordingly, AJ-treated plants showed a 16.4% reduction in malondialdehyde (MDA) content (*p* < 0.0001; Figure 9h), indicating alleviated oxidative damage. PAL and PPO activities also increased by 10.2% and 10.0% (*p* < 0.01; Figure 9f,g), reinforcing cell walls through phenolic and lignin synthesis [46] and generating antimicrobial quinones [47] Consequently, AJ application reduced gray mold incidence by 55% (*p* < 0.01; Table 5; Figure 8), indicating the induction of systemic resistance (ISR) with potential broad-spectrum protection [48].

While these biochemical changes clearly reflect activation of the antioxidant defense system, their biological relevance warrants critical evaluation. Increases in enzyme activity alone do not necessarily confirm functional resistance unless they correspond to real improvements in redox balance and disease suppression. Comparable studies in pepper demonstrated that concurrent elevation of SOD, CAT, and POD accompanied by decreased MDA correlated with delayed lesion development and reduced disease severity [49]. Moreover, WRKY transcription factors such as PnWRKY27 have been shown to regulate antioxidant gene expression, maintain ROS homeostasis, and strengthen systemic resistance [50]. These reports support that AJ-induced enzyme activation may contribute to enhanced oxidative tolerance and defense capacity in tomato.

It is also important to distinguish between resistance and tolerance when interpreting these responses. Resistance suppresses pathogen proliferation, whereas tolerance limits tissue damage under infection [51]. Since this study focused on host physiological changes rather than pathogen biomass, AJ’s effect likely reflects a tolerance-based defense, with plants maintaining membrane stability and growth despite infection. Nevertheless, the observed reduction in disease incidence suggests that both tolerance and partial resistance may be involved. Future studies quantifying pathogen load and ROS dynamics will help clarify this mechanism.

### 4.3. Implications for Sustainable Tomato Production

The dual mechanism of AJ, as established in this study, highlights its potential as a sustainable alternative to chemical fungicides in tomato production. Unlike synthetic fungicides, which pose risks of environmental degradation and pathogen resistance [11], AJ leverages natural organic acids and beneficial microbes, aligning with eco-friendly biocontrol strategies [12]. The study’s findings support AJ’s potential for field application, particularly given its low cost and use of agricultural byproducts [18]. However, challenges such as optimizing AJ concentration for large-scale use and ensuring consistent efficacy across diverse tomato cultivars and environmental conditions warrant further investigation. Future research should also explore AJ’s effects on other solanaceous crops and its integration into integrated pest management systems to maximize its practical utility.

Overall, the results demonstrate that AJ acts as a dual-action biocontrol product. It not only directly inhibits the pathogen but also primes the tomato plants’ physiological defenses, including enhanced structural barriers, antioxidant capacity, and cell wall reinforcement. This synergy between direct and indirect mechanisms effectively mitigates disease progression and underpins the observed reduction in gray mold incidence.

## 5. Conclusions

Agricultural Jiaosu (AJ) effectively mitigates tomato gray mold through a synergistic dual mechanism that integrates direct antifungal inhibition with the activation of host defenses. The acidic constituents of AJ rapidly disrupt the membrane integrity and redox homeostasis of *B*. *cinerea*, while microbial populations such as *Acetobacter* sustain suppression during later stages by competing for nutrients. In parallel, AJ enhances tomato systemic resistance by upregulating antioxidant and phenylpropanoid-pathway enzymes, thereby alleviating oxidative stress and restricting pathogen development.

Overall, the efficacy of AJ stems not merely from its acidity but from a coordinated interplay between biochemical suppression of the pathogen and physiological resistance of the host. As a low-cost, eco-friendly product derived from agricultural byproducts, AJ offers a promising biocontrol alternative to chemical fungicides and represents a valuable addition to sustainable tomato disease management strategies.

## Figures and Tables

**Table 1:** Primer sequence.

Primer	Sequence	Target
16S-F	5′-ACTCCTACGGGAGGCAGCA-3′	Bacteria
16S-R	5′-GGACTACHVGGGTWTCTAAT-3′	
ITS-F	5′-GGAAGTAAAAGTCGTAACAAGG-3′	Fungi
ITS-R	5′-GCTGCGTTCTTCATCGATGC-3′	

**Table 2:** Primer sequence.

Primer	Sequence	Target
F_alllact_IS	5′-TGGATGCCTTGGCACTAGGA-3′	*Lactobacillus* [30]
R_alllact_IS	5′-AAATCTCCGGATCAAAGCTTACTTAT-3′	
Ace-F	5′-GCTGGCGGCATGCTTAACACAT-3′	*Acetobacter* [31]
Ace-R	5′-GCTGGCGGCATGCTTAACACAT-3′	
Bc-F	5′-CAGGAAACACTTTTGGGGATA-3′	*Botrytis cinerea* [18]
Bc-R	5′-CAGGAAACACTTTTGGGGATA-3′	

**Table 3:** Organic acid concentrations in AJ.

Target	Concentration g L^−1^
Formic acid	1.39
Acetic acid	27.31
Propionic acid	7.62
Butyric acid	15.69
Lactic acid	5.99

**Table 4:** Volatile organic compounds (VOCs) of AJ.

No.	Retention Time (min)	Compound	Matching Degree	Area (%)
1	1.39	Acetic acid	86	23.51
2	9.79	L-alpha-Terpineol	90	6.78
3	0.28	Propanoic acid, ethyl ester	86	4.60
4	10.95	Cyclohexasiloxane, dodecamethyl-	94	3.97
5	7.03	Cyclotetrasiloxane, octamethyl-	91	3.13
6	1.85	Acetoin	86	2.71
7	9.63	3-Cyclohexen-1-ol, 4-methyl-1-(1-methylethyl)-, (R)-	96	2.36
8	10.49	2-phenylethyl ester	80	2.01
9	9.97	Creosol	94	1.36
10	12.77	bis(1,1-dimethylethyl)-	90	0.84
11	9.09	Phenylethyl Alcohol	93	0.79
12	7.60	D-Limonene	95	0.49
13	9.63	endo-Borneol	90	0.43
14	10.88	Phenol, 4-ethyl-2-methoxy-	83	0.35
15	8.86	Fenchol, exo-	93	0.33
16	13.88	2-Naphthalenemethanol,1,2,3,4,4a,5,6,7-octahydro-.alpha.,.alpha.,4a,8-tetramethyl-, (2R-cis)-	98	0.22
17	11.47	Benzenepropanoic acid, ethyl ester	91	0.18
18	6.36	4-Ethylbenzoic acid, heptyl ester	83	0.15
19	14.99	Cyclononasiloxane, octadecamethyl-	83	0.08

**Table 5:** Gray mold severity in tomato plants at 10 days after treatment with Agricultural Jiaosu (AJ).

Treatment	Number of Plants	Disease Severity Scale					Disease Index
		Score 0	Score 1	Score 2	Score 3	Score 4	
CK	20	0	0	0	4	16	95
AJ	20	3	8	4	2	3	47.5 ***

Note: The disease severity distribution is presented as plant counts per category. The disease index, calculated from all plants, was significantly different between treatments as per Chi-square test (*** *p* < 0.001).

## Data Availability

The original contributions presented in this study are included in the article/Supplementary Material. Further inquiries can be directed to the corresponding authors.

## Supplementary Materials

The following supporting information can be downloaded at: https://www.mdpi.com/article/10.3390/jof11120873/s1.
